# Exploring the potential for using results-based financing to address non-communicable diseases in low- and middle-income countries

**DOI:** 10.1186/1471-2458-13-92

**Published:** 2013-02-01

**Authors:** Chelsey R Beane, Suzanne Havala Hobbs, Harsha Thirumurthy

**Affiliations:** 1 Department of Health Policy and Management, Gillings School of Global Public Health, University of North Carolina at Chapel Hill, Campus Box 7411, 135 Dauer Drive, Chapel Hill, NC, 27599, USA

**Keywords:** Non-communicable diseases, Results-based financing, Pay-for-performance, Performance incentives, Health aid

## Abstract

**Background:**

The burden of disease due to non-communicable diseases (NCDs) is rising in low- and middle-income countries (LMICs) and funding for global health is increasingly limited. As a large contributor of development assistance for health, the US government has the potential to influence overall trends in NCDs. Results-based financing (RBF) has been proposed as a strategy to increase aid effectiveness and efficiency through incentives for positive performance and results in health programs, but its potential for addressing NCDs has not been explored.

**Methods:**

Qualitative methods including literature review and key informant interviews were used to identify promising RBF mechanisms for addressing NCDs in resource-limited settings. Eight key informants identified by area of expertise participated in semi-structured interviews.

**Results:**

The majority of RBF schemes to date have been applied to maternal and child health. Evidence from existing RBF programs suggests that RBF principles can be applied to health programs for NCDs. Several options were identified for US involvement with RBF for NCDs.

**Conclusion:**

There is potential for the US to have a significant impact on NCDs in LMICs through a comprehensive RBF strategy for global health. RBF mechanisms should be tested for use in NCD programs through pilot programs incorporating robust impact evaluations.

## Background

The current economic climate has led donor governments to reduce spending in all areas, including global health initiatives. Meanwhile, low- and middle-income countries (LMICs) are facing significant health challenges. Despite progress in some health indicators in the past 20 years, challenges from non-communicable diseases (NCDs) are increasing [1].

Increased funding for global health may be unlikely in the current economic climate. While numerous public and private entities are important funders of global health efforts, this paper focuses on the role of the United States government because of its influence on global health trends as the largest donor of development assistance for health (DAH). Currently, most US funding for global health is bilateral and disease-specific in nature, and finances program inputs with limited emphasis on health outcomes. These trends have prompted a renewed search for improvements in aid effectiveness and efficiency. While various financing strategies are currently under review, Results-Based Financing (RBF) mechanisms, which incentivize performance or results, have shown promising results in recent years [2,3].

RBF mechanisms have been applied to infectious diseases and maternal and child health, but very few have specifically focused on NCDs. Research in this area has also neglected RBF for NCDs. To date, the US has supported RBF for global health through the Global Fund for AIDS, Tuberculosis and Malaria, the Millennium Challenge Corporation (MCC), Health Systems 20/20, and USAID-supported projects. However, the US does not currently have an overall strategy for RBF [4]. As a major provider of DAH, the US has the potential to significantly impact NCDs in LMICs using RBF.

This study identifies specific RBF mechanisms, addresses their potential for use in NCD programs in LMICs, and presents options for US involvement. As few NCD programs to date have used RBF strategies, this analysis draws on evidence from existing RBF programs, which are mainly focused on maternal and child health and communicable diseases, and therefore addresses the challenge presented by the dichotomy between infectious diseases and NCDs that is common in global health. Furthermore, while this paper focuses on RBF specifically, it should be noted that RBF is part of a larger and more comprehensive strategy for addressing NCDs globally. This paper does not seek to examine the various other approaches to combatting NCDs.

### The rising burden of NCDs

While significant progress has been made in certain areas of global health [5,6], the burden of disease from NCDs – such as cardiovascular diseases, diabetes, certain types of cancers and chronic respiratory diseases – is rising. There were 36 million deaths due to NCDs in 2008 [5]. Tobacco use causes more deaths globally than HIV/AIDS, malaria, and tuberculosis (TB) combined, and 80% of those tobacco-related deaths are in the developing world [7]. NCDs cause about 56% of all deaths in LMICs and 46% of the disease burden in terms of disability-adjusted life-years [8]. Developing countries are facing higher morbidity and mortality from various types of cancer, some of which are attributable to infectious diseases [9]. The disease burden due to NCDs is expected to increase rapidly [8]. Deaths due to NCDs are projected to reach 55 million by 2030, up from 36 million 2008 [10]. Furthermore, evidence suggests that NCDs disproportionately affect the poor, cause poverty, and hinder economic development [11].

### Trends in development assistance for health

Until 2008, overall trends in DAH were positive, especially over the past decade [12]. The US is the largest donor of DAH in terms of absolute dollars; as a percentage of GDP, however, the US ranks 10th [13]. While US funding for global health increased from $4.4 billion in 2004 to $9.6 billion in 2008, this represented only .083% of US GDP [13,14]. There have been large increases in DAH, but only about 3% goes towards NCDs [15].

Most US funding for global health is channeled through the Global Health Initiative (GHI), created in 2009 to promote coordination for global health within the US government. While the GHI was authorized in 2009 for $63 billion over a six-year period, actual funding is determined annually by the US Congress. To curb US spending and reduce debt, funding for the GHI has been flat since 2009 at just under $9 billion annually in absolute terms [16]. Entering its fourth year in 2012, the GHI reached only 56% ($35 billion) of its originally proposed six-year budget [17].

Not only is US funding for global health becoming stagnant, but a recent report by the Center for Global Development ranked the US 29th out of 31 major donors on aid efficiency, with USAID ranking the lowest for maximizing efficiency when compared to five other US government agencies [18]. As the largest bilateral donor government for global health, US government contributions have a significant impact on overall trends. Given the growing challenges to global health and the uncertainty of resources, more efficient and effective DAH is urgently needed.

## Methods

Three RBF mechanisms were chosen for evaluation. Using literature review and key informant interviews the three were analyzed for potential use in strengthening NCD health programs. Mechanisms were chosen for their applicability to global health generally and NCDs specifically, and for their feasibility for use by the US government. Key informants were chosen for their expertise in global health, RBF and NCDs.

### Results-based financing mechanisms

A summary of RBF mechanisms considered for this study is provided in Table 1. The World Bank defines RBF as, “a cash payment or non-monetary transfer made to a national or sub-national government, manager, provider, payer or consumer of health services after predefined results have been attained and verified. Payment is conditional on measurable actions being undertaken.” [19].

**Table 1:** Inclusion/Exclusion of results-based financing mechanisms

**Mechanism**	**Definition**	**Inclusion/Exclusion criteria**
Output-Based Aid (OBA)	A results-based mechanism that is used to deliver basic infrastructure and social services to the poor	Excluded for use predominantly outside of the health sector
Cash on Delivery (COD)	A mechanism that gives recipients full responsibility and authority over funds paid in proportion to verified measures of progress toward a single specific outcome	Excluded for political difficulty in agent having complete control
Performance-Based Financing (PBF)	Fee-for-service paid to providers conditional on specific predefined indicators for degree and/or quality	Included for use in health sector and political feasibility
Performance-Based Contracting (PBC)	A fixed price for an output or outcome with a variable increase/decrease in payment based on performance, typically applied to NGOs	Included for use in health sector and political feasibility
Conditional Cash Transfers (CCTs)	A demand-side mechanism that provides incentives directly to program beneficiaries	Included for use in health sector and political feasibility

Source: World Bank [19], CGD [20].

Five main types of RBF are outlined by the World Bank, of which three were chosen for inclusion in this analysis: Performance-Based Financing (PBF), Performance-Based Contracting (PBC), and Conditional Cash Transfers (CCT). PBF and PBC are very similar. In PBF, payment is made only to providers (versus beneficiaries) and is typically financial in the form of fee-for-service. PBF is predominantly a supply-side intervention but can also incorporate demand-side incentives. Payment is stipulated by the degree or quality to which services are completed. PBC offers a fixed price for an output or outcome with a variable increase/decrease in payment based on performance. PBC is typically applied to non-governmental organizations (NGOs) [19]. Finally, CCTs are demand-side interventions that provide incentives directly to program beneficiaries and seek to motivate them to engage in certain desirable behaviors. CCTs offer both financial and non-financial incentives.

Policy levers, such as tobacco and alcohol taxes, regulation of the food industry, and efforts to reduce salt and sugar consumption play an important role in NCD prevention. However, such policies were excluded from consideration in this study, as they are likely to be the decision of governments in individual countries, may face significant policy obstacles, and necessitate minimal US government involvement. A comprehensive NCD strategy for global health, however, should consider RBF in addition to other interventions and policy efforts to achieve behavior change or address the risk factors for NCDs.

### Key informant interviews

An overview of key informant interviews is provided in Table 2. Interviews were used to collect data related to the experience, knowledge and opinions of key informants. Key informants were identified through a review of the literature and through the academic network at the University of North Carolina at Chapel Hill. Participants were selected using purposeful sampling. Ten experts who met the selection criteria of a minimum of ten years experience in global health and with expertise in one or more of RBF, PBF, PBC, CCTs, or NCDs were selected and contacted via email by CRB to participate in the study.

**Table 2:** Characteristics of key informant participants

**Item**	**Number**
Key informants identified (#)	10
Total participants (#)	8
Organizations represented (#)	7
Avg. experience in global health (years)	24
Participants with expertise in RBF (#)	6
Participants with expertise in NCDs (#)	4

Of ten identified experts, eight participated in semi-structured interviews (two did not respond during the study period). Information about the study was provided to participants and consent to participate was received via email before data collection as well as verbally at the time of the interviews. Participants represented seven organizations and had an average of 24 years of experience in global health, with experience in every region of the world. Each participant was interviewed by CRB concerning RBF, NCDs, and US involvement. Interviews were conducted by telephone or Skype between January and March 2012. Data collection ended after interviews were conducted with all participants. Interviews were semi-structured and used an interview guide developed by CRB. Interview questions were open ended and interviews were recorded and detailed notes were taken. Where possible, secondary data were used to complement data collected from key informant interviews.

Interview responses were systematically categorized and thematic analysis was used to identify and describe themes.

## Results

### Experience of RBF to date

The evidence from RBF for health is mixed. Given the diversity of RBF schemes, there is significant variability between projects. The design of RBF programs is highly context-specific. Health indicators and performance incentives are largely dependent upon the specific health needs and risk factors of the population, the health system and infrastructure in the country, and the priorities of stakeholders. Limited and/or inadequate monitoring and evaluation further complicate efforts to review RBF programs. A Cochrane review of the effects of paying for performance in health care in LMICs concluded that more robust and comprehensive studies are needed, a finding supported by other studies of RBF as well [21-24]. More extensive monitoring and evaluation is particularly necessary for showing results in the long-term [25]. Despite the need for additional evidence, it has been suggested that RBF can be an important tool for addressing efficiency and accountability, as well as health sector reform more broadly [26].

Rwanda has perhaps the best-known and most rigorously evaluated RBF program. Evidence from Rwanda is promising. The impact evaluation of Rwanda’s PBF program determined the effect on fourteen maternal and child health indicators [27]. While some indicators showed more positive results than others, overall results demonstrated that RBF can help to increase service delivery and quality of care, improving health sector performance [28]. Various other PBF programs have demonstrated positive or mixed results, such as PBF schemes in the Democratic Republic of Congo, Egypt, and Burundi [29-31]. Less successful programs, such as the Tanzania PBF scheme, have provided important lessons [32].

PBC programs, like PBF, have generally produced encouraging results. A PBC scheme in Cambodia found that contracting NGOs was effective in increasing service delivery [33]. Promising results have also come from other countries, such as PBC programs in Liberia and Afghanistan [34,35]. A PBC scheme in Southern Sudan, however, experienced significant implementation challenges [36], and in Uganda various survey rounds found no impact from PBF schemes. Lessons from Uganda include the need for substantial enough incentives directly tied to results, autonomy for health facilities to make decisions and to use resources, and rigorous verification of results [37].

*Progresa/Oportunidades* in Mexico is the largest and most cited CCT program. This program provided incentives to households in the form of cash transfer payments to women conditional on engaging in certain behaviors related to child health, nutrition and education. Evaluation has shown success in improving child health indicators, among other areas [38]. A similar program in Brazil, *BolsaFamilia,* however, produced no positive results in terms of health status, but succeeded in reducing poverty, suggesting that demand-side incentives alone may not be enough to improve health outcomes [39,40]. Other CCT programs, such as the Janani SurakshaYojana program in India, have shown positive yet mixed results [41]. Psychological research has demonstrated in clinical trials that incentives can be effective in changing behaviors related to smoking and drug use as well, however, these findings must also be proven in population-based interventions [42].

While both PBF and PBC programs have produced mixed results, on the whole there is much encouraging evidence. Demonstrated successes from some programs and lessons taken from others, suggest that both of these approaches could be applied to NCDs. CCT programs, have been successful at reducing poverty, which has important health implications. CCT programs could have potentially significant effects on risk factors for NCDs, including diet, exercise, and tobacco use, as well as preventive measures for certain types of cancers. The benefit of using supply-side interventions in tandem with demand-side incentives, suggests that the use of CCTs for NCDs may be most effective when combined with other supply-side programs.

### Applicability of RBF to NCDs

While most RBF programs have focused on Millennium Development Goal (MDG)-related health issues, usually on maternal and child health (MCH), a few have included NCDs. In Belize, a PBF program designed to scale up access and improve quality of health services targeted chronic illnesses in addition to prenatal and postnatal care. Evaluation of the six month pilot showed an increase in use of primary care and the diagnosis and treatment of diabetes and hypertension, the country’s top two causes of death [43,44]. In Abu Dhabi, the Weqaya (or prevention) program has used performance incentives for NCDs to improve quality and measurable health indicators through the use of Disease Management Programmes (DMPs) to address compliance with clinical care and behavior change related to specific risk factors at the patient level. As the program was initiated in 2006, the long-term effects are yet to be seen [45].

Few RBF-NCD programs may have been initiated because the epidemiological nature of NCDs does not easily lend itself to RBF. Programs aimed at NCDs often involve major health behavior change and long-term interventions. Incorporating RBF into these programs is more difficult, compared to one-time or short-term interventions, such as immunizations [46]. A RBF expert from the World Bank made the distinction that RBF focuses on both health outputs and outcomes. It is more challenging, however, to have specific measurable indicators for health outcomes that may occur over long time periods, which is the case for many NCD indicators. This is due to the need to provide incentives on the supply-side on a regular basis (i.e. monthly or quarterly) when using PBF. Therefore, incentives to health facilities are often linked to more short-term outputs, such as immunizations, antenatal care, institutional deliveries, growth monitoring, and family planning. For example, a NCD health intervention focused on tobacco use might use smoking prevalence as measured by population-based survey data as an indicator. Tying incentives to percentage decreases in smoking prevalence, however, would be difficult. Instead, it might be more effective to use the number of people enrolled in smoking cessation programs as an indicator, allowing incentive payments to providers to be made quarterly.

Rena Eichler and Ruth Levine note the potential of RBF for NCDs based on evidence from developed-country settings [46]. For example, a supply-side intervention for diabetes in the United States demonstrates the potential of performance incentives for chronic conditions [46]. Demand-side incentives have been used to effect behaviors to reduce consumption of tobacco and alcohol but have not been successful in the long-term [46]. The minimal available evidence suggests that supply-side RBF programs for NCDs are likely to be more effective than demand-side programs.

Although little evidence is available on RBF for NCDs, analysis of RBF programs for MCH, communicable diseases, and health systems strengthening (HSS) provides a reasonable basis for applying RBF concepts to NCDs. NCD programs would benefit from improvements in service delivery, quality, and health sector performance that may result from RBF programs directed at MCH or HSS [3]. For example, improved access to essential medicines and primary care could mean increased adherence to chronic disease medications and increased rates of screening for certain types of cancers. Improved health care quality could include indicators for health IT, which could in turn improve chronic disease management. Broader health system effects from RBF could have meaningful implications for NCDs.

Despite many challenges, application of RBF principles to NCDs has great potential. A summary of common themes from key informant interviews can be seen in Table 3. Expert testimony highlighted the potential of RBF and cautioned about the challenges, such as the long-term effects of NCDs and health behaviors external to the health system. Even these behaviors such as diet and exercise, may lend themselves to incentive programs. The potential for RBF, key informants noted, depends on country-specific priorities and risk factors. An expert on RBF from the World Bank saw potential in using RBF for preventive and health promotion services, two areas into which NCDs fall. It is important to integrate NCD indicators into existing RBF schemes where possible. There is also potential for linking efforts to reduce NCDs to a RBF mechanism as part of a larger package of essential health services. Kathy Kantengwa, an expert on PBF from MSH, stated that RBF can apply to any service delivery, and therefore could be used for early detection of NCDs and management. She stated:

*“The RBF principles for communicable disease can be adapted to the NCDs”*[47].

**Table 3:** Common themes from semi-structured interviews

		
1	Key elements of successful RBF programs	Political commitment, government ownership, buy-in of stakeholders
		Clearly defined rules, understanding of indicators; accountability, verification of indicators
		Measuring and evaluation
		Design of program, piloting and testing; participatory approach
		Flexibility in implementation; communication, transparency, sustainability
2	Areas of health for which RBF traditionally used	Maternal and child health; MDGs 4 and 5
		Health service delivery, primary care, quantity and quality of services
3	Potential use of RBF for NCDs	Application of RBF to any service delivery
		Incentivizing preventive and health promotion activities; national, institutional, and individual levels
		Part of package of essential health services; combining efforts for communicable and non-communicable diseases
4	Challenges in taking a RBF approach	Variation in capacity of donor agency representatives
		Use of RBF as panacea, depletion of resources; unintended consequences
		Insufficient ownership and accountability; corruption
		Technical assistance-intensive to establish new/sustainable systems
		Complexity of RBF; significant time for design and implementation
		Skepticism about RBF mechanisms
5	Potential for US involvement with RBF for NCDs	Collect best practices from RBF; assess epidemiological situation
		Engage stakeholders; take participatory approach
		Pilot programs to test applicability of RBF for NCDs; increase funding for NCDs
		Include NCDs as part of package of essential health services; avoid dichotomy between communicable and non-communicable diseases

The challenge for NCDs arises in determining which services need to be purchased, how and by whom, as well as how to measure the results. Patricio Marquez, Lead Health Specialist at the World Bank suggested using existing RBF programs to identify the entry points to cover some of the interventions that may have an impact on the onset of NCDs [48].

Experts on NCDs identified various approaches for utilizing RBF mechanisms. Rachel Nugent, an expert on NCDs from the University of Washington, noted that the need for patient involvement in managing NCDs lends itself to incentive programs for both prevention and treatment related to NCD risk factors. Incentives could be provided to eat and exercise correctly, for tobacco cessation, or for adherence to medication [49]. Patricio Marquez suggested using HIV programs as an opportunity to target certain forms of cancer, using maternal health schemes to also affect cervical cancer or using TB interventions as the entry point for tobacco control programs [48]. Other examples of NCDs with infectious components include Hepatitis B and liver cancer, diabetes and TB, and lymphoma and malaria [50]. An important aspect of MCH programs is childhood malnutrition, a leading risk factor for NCDs such as diabetes. Given the interconnectedness of communicable diseases, MCH and NCDs, health intervention efforts could be complementary. In the words of Patricio Marquez:

*“It’s time to start thinking of the patient as a whole. Let’s stop seeing the patient or populations by disease. Because the interconnection between communicable and non-communicable diseases is there. So the question is how we adopt a global health strategy that avoids the dichotomy. By doing that we will be able to prevent the proliferation of government programs that in some cases create distortions”*[48].

Other key informants stressed the importance of taking a policy-level approach, often in addition to individual-level interventions. Dr. Sameh El-Saharty, a Senior Health Policy Specialist at the World Bank, listed several interventions that could benefit from a RBF strategy including banning tobacco advertisements and smoking in public places, encouraging exercise and seatbelt use, and incentivizing industries to use healthier foods. At the institutional or sub-national level the same mechanisms could be used to incentivize institutions to adopt programs or revise procedures, such as clinical protocols or health insurance programs, and to introduce screening programs for conditions such as cancer and cardiovascular disease. RBF could be used at the sub-national level to incentivize states, regions, or districts, or on the supply-side to provide incentives at the service delivery level [51]. From supply-side to demand-side, national-level to individual-level, and NCD-focused to basic health service-focused programs, experts agreed that RBF could be used to affect NCDs in resource-limited countries.

### Key elements of successful RBF programs

Experience from RBF programs has provided lessons useful when designing NCD programs using RBF mechanisms. RBF experts specified numerous key factors in designing and implementing successful RBF programs, such as political commitment, a participatory approach, clearly defined rules and targets, strong verification systems, and robust measuring and evaluation. A summary of these themes can be seen in Table 3. One expert on RBF from the World Bank said:

“RBF is so different from traditional input-based financing, in order for it to work, you need political commitment, and you need commitment from technical levels of Ministry of Health and Ministry of Finance, to be able to make the whole thing work. People really have to be convinced… And that’s not unique to RBF, but it’s more important in RBF because it’s such a different system.”

Various other elements were stated as being key to developing a successful program. Testing mechanisms on a small scale through pilot projects is crucial in identifying potential challenges early on. Joseph Naimoli, an expert from USAID/CDC, stated:

*“They spend a considerable amount of time designing these programs and getting them right. And also, they spend some time in piloting and testing these things in a smaller geographic area – like at the district level or multiple districts – and spend some time understanding what the potential obstacles are and trying to work those obstacles out. I think those kinds of programs are the most successful”*[52].

Flexibility in implementation and the ability to adapt to context-specific conditions, having basic health infrastructure in place, and developing open communication are also central.

## Discussion

### US involvement

Although the US has not yet formed a comprehensive RBF strategy for DAH, it supports various RBF programs. Currently, USAID supports countries both directly and indirectly through the funding of technical partners. In these ways, USAID has supported RBF programs in Rwanda, Pakistan, Ethiopia, Egypt, South Africa, Southern Sudan, Cameroon, and Haiti, among others. RBF could be an important mechanism in the broader global health agenda of value for money, which is being addressed by the Center for Global Development, the UK’s Department for International Development (DFID), and the Global Fund for AIDS, Tuberculosis, and Malaria, among others.

Donor governments have used RBF to promote health, increase aid effectiveness and improve outcomes. They have the ability to use their resources to encourage providers, patients, and systems to act in certain ways to achieve specific outcomes [46]. An expert from USAID/CDC, stated that the first goal of RBF for the US government is health outcomes related to MCH per MDGs 4 and 5, and HSS as outlined by the WHO building blocks [52]. Funding and budget considerations also play a role, as well as aid effectiveness. An expert on PBF from MSH observed:

*“The development assistance is from the people, so they want to know that each dollar spent is adding value and is really reaching the results or the outcomes that they are meant for”*[47].

The US government, through its global health entities, can take a variety of steps to make the most appropriate use of RBF and to address the growing NCD burden in LMICs countries. Four specific measures are offered for consideration below.

1. Collect best practices. US government agencies should review the best practices and lessons learned from existing RBF programs. This effort can build on the Health Systems 20/20 pay-for-performance (P4P) Case Study Series. By understanding the experience with communicable diseases, MCH and HSS, RBF programs for NCDs can avoid known pitfalls and have the greatest possible impact. The risk factors for NCDs and effective prevention and treatment strategies have been well documented. The best practices for reducing NCDs in resource-limited settings should also be reviewed and utilized.

2. Access epidemiological data in countries where the US already has large development assistance programs. Identify the NCD risk factors and potential areas that should be targeted for health interventions. Target specific NCDs that are linked with certain communicable diseases or incorporate NCD components into existing primary care and health system strengthening efforts.

3. Take a participatory approach and engage stakeholders. The best results may arise through being pragmatic and flexible, and encouraging buy-in from US representatives, partner country governments, program managers, health workers and providers, and other key stakeholders.

4. Invest in pilot programs. Only through testing the applicability of RBF for NCDs will a thorough understanding of its potential effects be gained. Incorporating robust monitoring and evaluation of pilot programs will strengthen the body of evidence for RBF as a whole, and provide preliminary evidence for the use of RBF for NCDs.

There are a few additional strategies to consider while engaging in the above options with regard to RBF for NCDs. One is to include NCDs as part of a larger package of health services. As has been demonstrated in other areas, taking a vertical approach with RBF can have unintended consequences, such as the diversion of resources, perverse incentives among providers and within the health system, and corruption. Another is to increase funding allocated for NCDs. The majority of US DAH today is earmarked for infectious diseases. As NCDs continue to increase throughout the developing and developed world, the current distribution of funds will need to be altered. Incorporating NCDs into current global health programs is one way to bridge this gap.

It should also be noted that other measures not specifically highlighted in interviews, such as the need to work closely with other donor governments and multilateral entities to incorporate RBF for NCDs into existing health programs and RBF schemes, are also important. Finally, any RBF mechanism used for NCDs should be part of a much broader strategy for reducing NCDs in LMICs. Such a strategy could include efforts to promote lifestyle changes to address the risk factors for NCDs both domestically and internationally, as US national efforts have the potential to influence trends globally. By considering multiple strategies and using best practices for prevention and treatment, there is potential for the US to be a leader in the global fight against NCDs.

### Limitations

The context-specific nature of this study limits the ability to generalize results, or to make concrete conclusions about the role of RBF for NCDs. Furthermore, as most RBF programs have been focused on MCH and communicable diseases, there is little evidence specific to RBF for NCDs. To increase reliability and rigor, a larger number of key informant interviews with greater variety in areas of expertise would have been beneficial. Due to time and resource constraints, the number of key informant interviews was limited to eight.

Limitations to the qualitative interviews include selection and response bias. Key informants were intentionally recruited to represent a variety of organizations and areas of expertise. The two non-respondents may have revealed unrepresented viewpoints on RBF and NCDs; however, the goal of the interviews was to identify themes based on expert opinion, which was satisfactorily accomplished. The researchers’ influence on the formulation on the research question, data collection and analysis introduces the possibility of research bias. However, data collection and interpretation focused on the identification of themes and did not form judgments on interviewee responses. To further reduce bias the interview guide was reviewed by the research team and one unaffiliated third party. Interview notes were also checked with recordings for accuracy of interpretation.

The lack of peer-reviewed literature on RBF program evaluations/impacts limited the amount of information available through systematic review. The majority of the literature review was derived from World Bank Results-Based Financing for Health documents and the USAID Health Systems 20/20 P4P Case Study Series.

Finally, this analysis was designed to present options for US government involvement, and therefore does not address specific options for other government actors or private sector involvement. More research on use of RBF in the private sector and among other government actors and global health entities and possibilities for the use of these strategies for NCDs is warranted.

## Conclusion

Research findings suggest that RBF mechanisms may be effectively used to positively affect NCD prevention and control in resource-limited settings. Both PBF and PBC schemes have shown promising results in programs focused on MCH, HSS and communicable diseases, and CCT programs have important implications for health outcomes through poverty reduction. Evidence and lessons learned from these programs provide the basis for designing and piloting RBF programs for NCDs. While this evidence provides a basis, rigorous monitoring and impact evaluation of new and existing RBF programs is necessary to continue building the body of evidence.

It is important to emphasize that RBF is one of several mechanisms that countries such as the US can use to combat NCDs in LMICs. These could include efforts by the US to promote behavior change in LMICs. Incentives for individuals have been shown to result in behavior change and such programs could also receive greater consideration [53,54]. In addition, some of the most effective strategies for combatting NCDs may not lend themselves to RBF mechanisms. Therefore, all potential strategies should be considered and the best practices for reducing NCDs should be utilized, incorporating RBF mechanisms where appropriate and as part of a larger comprehensive NCD strategy.

### Implications for practice

Given the significant amount of DAH from the US government and the great influence the US has on global health priorities, the US has the ability to significantly affect NCDs in LMICs. Developing a comprehensive strategy for the use of RBF within the US government global health agencies could serve to improve health performance and use aid more efficiently.

Given the increasing challenges and burden of disease from NCDs in LMICs, NCDs are a top priority area for global health. RBF principles can be applied to NCD programs, taking into account the evidence currently available from existing RBF schemes. The US has a number of options available to effectively examine the applicability of RBF for NCDs, and to incorporate RBF as part of a broader NCD strategy to have a significant impact on global health.

## Competing interests

The authors declare that they have no competing interests either financial or non-financial.

## Authors’ contributions

CRB developed the study, wrote the first draft, and made revisions; HT and SHH provided input on study methods, reviewed the first draft and subsequent versions. All authors read and approved the final manuscript.

## Pre-publication history

The pre-publication history for this paper can be accessed here:

http://www.biomedcentral.com/1471-2458/13/92/prepub
